# Advances in Nanocarriers for Effective Delivery of Docetaxel in the Treatment of Lung Cancer: An Overview

**DOI:** 10.3390/cancers13030400

**Published:** 2021-01-22

**Authors:** S. Aishah A. Razak, Amirah Mohd Gazzali, Faisalina Ahmad Fisol, Ibrahim M. Abdulbaqi, Thaigarajan Parumasivam, Noratiqah Mohtar, Habibah A. Wahab

**Affiliations:** 1 School of Pharmaceutical Sciences, Universiti Sains Malaysia, Minden, Penang 11800, Malaysia; sitiaishah.ar@student.usm.my (S.A.A.R.); faisalina@nibm.my (F.A.F.); ibrahimm.abdulbaqi@student.usm.my (I.M.A.); thaigarp@usm.my (T.P.); noratiqah@usm.my (N.M.); 2 Malaysian Institute of Pharmaceuticals and Nutraceuticals (IPharm), National Institute of Biotechnology Malaysia (NIBM), Ministry of Science, Technology and Innovation (MOSTI), Gelugor, Penang 11700, Malaysia

**Keywords:** docetaxel, nanoparticles, lung cancer, drug delivery, non-small cell lung cancer (NSCLC)

## Abstract

**Simple Summary:**

As lung cancer has the highest incidence rate compared to any other type of cancer, there have been extensive research studies aiming at finding a better treatment for curing this disease. One of the many approaches is by improving the delivery of anticancer drugs towards cancer cells using advanced technologies. In this review, we focused on docetaxel as one of the most commonly used drugs for lung cancer treatment and discussed the properties of the drug and the application of nanotechnology in delivering this drug to improve its efficacy and specificity while reducing its side effects.

**Abstract:**

Docetaxel (DCX) is a highly effective chemotherapeutic drug used in the treatment of different types of cancer, including non-small cell lung cancer (NSCLC). The drug is known to have low oral bioavailability due to its low aqueous solubility, poor membrane permeability and susceptibility to hepatic first-pass metabolism. To mitigate these problems, DCX is administered via the intravenous route. Currently, DCX is commercially available as a single vial that contains polysorbate 80 and ethanol to solubilize the poorly soluble drug. However, this formulation causes short- and long-term side effects, including hypersensitivity, febrile neutropenia, fatigue, fluid retention, and peripheral neuropathy. DCX is also a substrate to the drug efflux pump P-glycoprotein (P-gp) that would reduce its concentration within the vicinity of the cells and lead to the development of drug resistance. Hence, the incorporation of DCX into various nanocarrier systems has garnered a significant amount of attention in recent years to overcome these drawbacks. The surfaces of these drug-delivery systems indeed can be functionalized by modification with different ligands for smart targeting towards cancerous cells. This article provides an overview of the latest nanotechnological approaches and the delivery systems that were developed for passive and active delivery of DCX via different routes of administration for the treatment of lung cancer.

## 1. Introduction

Among all types of cancers, lung cancer is the most commonly diagnosed in both males and females globally and also the leading cause of cancer-related deaths [1]. Research studies are continuously carried out to find a better solution in treating lung cancer as the current treatment regimen often associated with nonspecific and nonselective cytotoxic chemotherapy or surgery. There are several classes of drugs that have been approved by the United States Food and Drug Administration (US FDA) to treat lung cancer, and among the commonly used ones is the antineoplastic taxane: docetaxel (DCX). 

**Figure 1:**
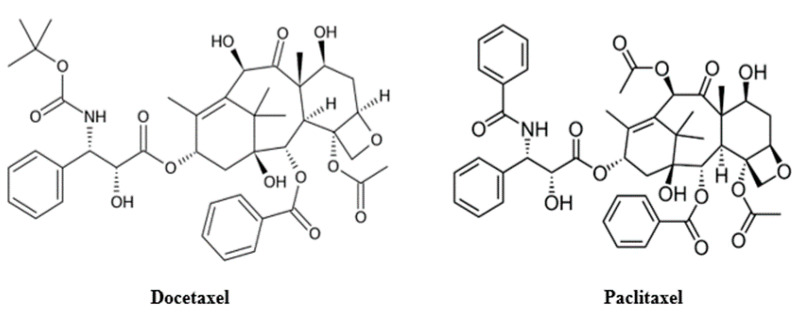
Chemical structure of docetaxel (**left**) and paclitaxel (**right**).

Docetaxel, discovered by Pierre Potier in National Center for Scientific Research in France during the 1980s, belongs to the taxoid class of cytotoxic agents together with paclitaxel (PCX) [2]. In 1971, paclitaxel (taxol) was identified as the active compound of the crude extract of the bark of the Pacific Yew tree *Taxus brevifolia.* Due to the limited supply of the drug from the natural product, there was a race to improve the production or find a new synthetic route of PCX. While working on the efficient partial synthesis of PCX from its congener, 10-deacetylbaccatin III [2], Portier discovered docetaxel. 10-deacetylbaccatin III has also been reported to be isolated from other members of the *Taxus* family (e.g., *T. baccata* and *T. brevifolia)* trees [3]. DCX has a structure similar to paclitaxel except for its tert-Butyl carbamate ester in the side chain of the phenylpropionate and the hydroxyl functional group on carbon-10 (Figure 1). The difference in their structures makes DCX slightly more soluble in water than PCX [4].

DCX was first approved by the US FDA for the treatment of breast cancer in 1996. It was also tested in the clinical trials for use the in treatment of non-small cell lung cancer (NSCLC) by Aventis Pharmaceutical Inc., Paris, France, (now Sanofi) as an intravenous formulation Taxotere^®^. Later, it was approved for the treatment of NSCLC in patients with locally advanced or metastatic NSCLC upon failure of platinum therapy as a single agent in 1999. In 2002, Taxotere^®^ was also approved for the treatment of locally advanced or metastatic untreated NSCLC with cisplatin. In addition to lung cancer, its use has been indicated for squamous cell cancer of the head and neck, gastric adenocarcinoma, breast cancer and prostate cancer [5] due to its cytotoxic effect on microtubules [6].

The cytotoxic effect on microtubules originates from the mechanism of DCX that inhibits cell proliferation by inducing a sustained block at the metaphase-anaphase boundary during cell division, thus disrupting the microtubular network that is significant for mitotic cell division [7]. DCX also inhibits the depolymerisation of the microtubule back to tubulin that leads to the failure of cell division and eventually, cell death [8]. Since DCX affects cell division, the drug is not only cytotoxic to cancer cells but also cytotoxic to the hair follicles, bone marrow and other germ cells. Thus, patients administered DCX frequently exhibit chemotherapy side effects that include hair loss. Furthermore, DCX has high plasma protein binding (>98%), which requires the administration of high doses in clinical settings. In some reports, the issuance of DCX at a high dose (>75 mg/m^2^) for the treatment of cancer, NSCLC, has produced side effects such as neutropenia, asthenia, neuropathy, and others [9]. The high dose barrier can be mitigated if the drugs are designed to be more site-specific and more targeted as opposed to the current conventional intravenous (IV) delivery. For instance, targeted nanohybrids based on the titanate nanotubes incorporated with DCX showed enhanced cytotoxicity against human PC-3 prostate adenocarcinoma cells and less toxic than the free DCX in vitro [10]. Similarly, a cocktail administration of DCX and a photosensitizing agent incorporated in hyaluronic acid-coated nanoparticles improved the intracellular drug concentration with a concomitant slow-release inside the human breast cancer cells as compared to the free drug group treatment group [11]. These findings signify that the hybridization of DCX with nanotechnology is a promising approach to mitigate the dose-related adverse effect of DCX. Hence, this review aims to provide an exhaustive overview of the current nanotechnological advances that utilized various nanoparticle platforms and DCX for effective treatment of cancer.

## 2. Physicochemical Properties of DCX

DCX is a white to off-white powder that is typically crystalline in nature. It has a molecular formula of C_43_H_53_NO_14_ and molecular weight of 807.89 Da. The melting point of DCX is 232 °C. For every drug, the most crucial physicochemical properties to be considered are the aqueous solubility and membrane permeability, as explained by Lipinski’s rule [12]. DCX has a partition coefficient (log-P) value of 4.1 and p*K*_a_ of 10.97 [13] which result in a low aqueous solubility (0.025 μg/mL) and a low membrane permeability (1 cm/s × 10**^−^**^6^). Hence, DCX is classified as Class IV of the biopharmaceutical classification system (BCS) [14].

## 3. Pharmacokinetics (PK)

The pharmacokinetic (PK) profile of DCX was consistent with the three-component PK model in which the half-lives for the alpha, beta and gamma phases were 4.5 min, 38.3 min, and 12.2 h, respectively [15]. Currently, the standard dose of DCX is between 75 and 100 mg/m^2^ and varies dependent on the type of cancers and the treatment available [16]. In the human body, the drug is distributed from central to the peripheral compartment at a total volume of distribution of 22 L/h/m^2^ and a mean stationary distribution volume of 113 L, depending on the liver function, age, body surface area, and plasma protein [4].

The current route of administration is intravenous. Following the administration, DCX will accumulate to a greater extent at the liver, bile ducts, muscles, pancreas and stomach. Moreover, the drug deposition is evidently high at cancerous cells compared to healthy cells as DCX binds extensively to α-1 acid glycoprotein (AAG) [17] in addition to the other plasma proteins such as albumin and lipoproteins. AAG is expressed significantly at a high level in cancer cells, hence becoming the central determinant in evaluating variability in serum binding as well as clearance of DCX from the body. DCX has been reported to be unbound for about 4% to 10% in the plasma of the patients that are treated with DCX, which indicates that DCX can bind extensively to the proteins [16].

DCX undergoes hepatic metabolism mainly by cytochrome P450 (CYP) 3A isoforms CYP3A4 and CYP3A5. The resulting metabolites and the parent drug are eliminated from the body predominantly via biliary and intestinal excretion [18,19] with the excretion in the faeces mainly as metabolites. DCX metabolic transformation was considered to be a detoxification pathway because the metabolites showed a marked reduction in cytotoxic activity against several cell lines compared to the parent drug [20]. Several studies have investigated the effect of cigarette smoke on the metabolism of anticancer drugs including docetaxel [21]; however, some evidence has pointed out that cigarette smoking does not alter the pharmacokinetic determinants of DCX and PCX, although smokers treated with DCX and PCX have less neutropenia and leukopenia [22].

### 3.1. Mechanism of Action of DCX in Lung Cancer

DCX, like PCX, inhibits depolymerization and disassembly of microtubules by binding to and stabilizing tubulin to cause cell-cycle arrest in G1/M phase, which leads to cell death. The anticancer effect of DCX is exerted by selective binding to β-subunit of polymerized tubulin to promote polymerization that will disrupt the assembly of microtubules and at the same time inhibit their depolymerization [23]. Since DCX will bind strongly to tubulin and promotes their polymerization, the stability of microtubules will be distorted and cause the mitotic cell cycle to be interrupted, leading to cell death. Although DCX shares the same mechanism as PCX, DCX is twice as potent as PCX in its ability to inhibit depolymerization. It has a higher binding affinity to tubulin, which makes it more effective in inhibiting cancerous cells compared to PCX [24]. 

In addition to the usual mechanism in inhibiting the cell cycle, DCX also offers clinical advantage through its association with b-cell-lymphoma-2 (BCL-2). BCL-2 family proteins play a key role in the intrinsic death pathways [25] and have anti-apoptotic and pro-apoptotic properties. Studies have shown that BCL-2 overexpression enhances in vitro sensitivity to DCX in NSCLC [26,27]. In addition, DCX has been reported to have an antiangiogenetic effect [28,29], and the ability to induce pro-inflammatory genes and proteins including tumor necrosis factor-α, various interleukins and enzymes such as nitric oxide synthase and cyclooxygenase-2 [30].

### 3.2. DCX Resistance

**Figure 2:**
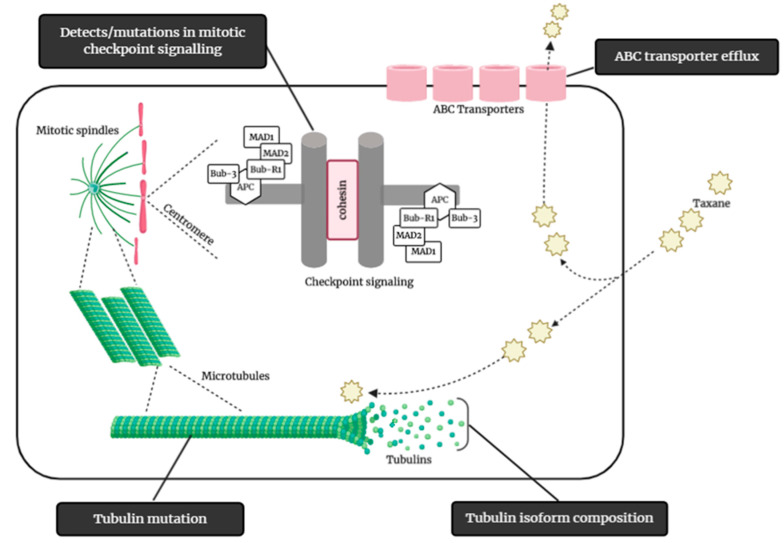
Some of the possible mechanisms of taxane resistance, such as modification of tubulin isoform composition, mutation of tubulin, mitotic checkpoints signaling mutation/defects, and ABC transporter efflux of taxane. (Illustrated through Biorender.com).

Drug resistance is a major cause of therapeutic failure in NSCLC, leading to tumor recurrence and disease progression. Various cellular mechanisms that give rise to resistance to taxanes, including DCX, have been identified (Figure 2). These include active efflux of the drug from the tumor cell, modification of drug targets, changes or mutation in β-tubulin subunits of microtubules, drug sequestration, detoxification of cytotoxic agents, and enhanced DNA repair mechanisms [31].

The mutation in β-tubulin isotype III or IV affects the binding or any post-transcriptional modification in β-tubulin and leads to structural change in microtubules, causing the resistance to develop [32]. DCX can cause cycle arrest at the G2/M phase but the odds of killing all the cancer cells is low. A few of the cancer cells will survive and enter multinucleated polyploids that express CD44 and develop a resistance to DCX. CD44 achieved the resistance to DCX by binding to osteopontin—an inflammatory cytokine that is related to metastatic progression. This interaction provides a feedback loop for the survival of the dispersed cells that resembles the origin of the cancer stem cells [33]. 

The most important barrier in delivering DCX to tumour cells is the drug efflux pump P-glycoprotein (P-gp) that contributes to multidrug resistance. P-gp is an ATP-binding cassette transporter and it is distributed throughout the intestinal epithelia, hepatocytes, kidneys, and capillary endothelial cells. P-gp is usually expressed on the apical side of epithelial cells of the trachea and major bronchi of the normal lung [34]. The activity of P-gp is prompted by endogenous lipids and peptides or by drugs that are substrate to it. DCX unfortunately is a substrate of P-gp, where it could cause dose-dependent activation of ATPase that gradually decreases the bioavailability of DCX [35]. Although P-gp is widely investigated and known for its contribution in the development of multidrug resistance, in lung cancer however, the role of P-gp overexpression in chemoresistance has been inconsistent. Merk et al. examined various transporters related to drug resistance, including P-gp, but they found no correlation of overexpressed P-gp and chemo-sensitivity [36]. Contradictorily, Chiou et al. reported the opposite as they did found positive correlation between P-gp and the reduction in DCX activity [37].

The tumor microenvironment consists of the tumor’s vasculature, connective tissue, infiltrating immune cells, stromal fibroblast and various bone-marrow-derived cells including macrophages, myeloid-derived suppressor cells and others [38]. The resistance to DCX contributed by tumor microenvironment might be through the paracrine amplification loop of various cytokines and growth factors produced by the stroma and cancer cells adhesion to the extra-cellular matrix. Other factors in the tumor microenvironment that will lead to drug resistance include the presence of overexpressed growth factors such as vascular endothelial growth factor and cytokines including interleukin-6 (IL6) and nuclear factor-κB (NF-κB) [39].

## 4. Drug Delivery for DCX

### 4.1. Route of DCX Delivery

The oral delivery of DCX is challenging because of low bioavailability, extensive first-pass metabolism and P-gp efflux pumps, as mentioned above. Thus, the intravenous (IV) route is commonly used for DCX delivery whereby the drug solution is injected through the vein and will instantly circulate in the bloodstream throughout the whole body. The physicochemical properties of the drug/carrier delivered are important as they will determine the efficacy of the drug. Particles with a size larger than 500 nm are not suitable to be administered via IV as they will be cleared rapidly from the circulation. The particles to be delivered to the tumor site should ideally have a size that is lower than 200 nm with a spherical shape and smooth texture for the ease of transportation through tumor vasculature [40]. Compared to other routes of delivery (i.e., oral, intraperitoneal, transdermal and rectal), parentally administered DCX demonstrated a higher area under curves (AUC) in the plasma. However, it will also lead to a high accumulation of DCX in the liver and heart after 15 min of administration [41]. Although IV routes provide flexibility and versatility in the treatment process, the administration will expose the healthy organs to the cytotoxicity and side effects of the drug. Thus, localized therapy for targeted delivery should be considered.

**Figure 3:**
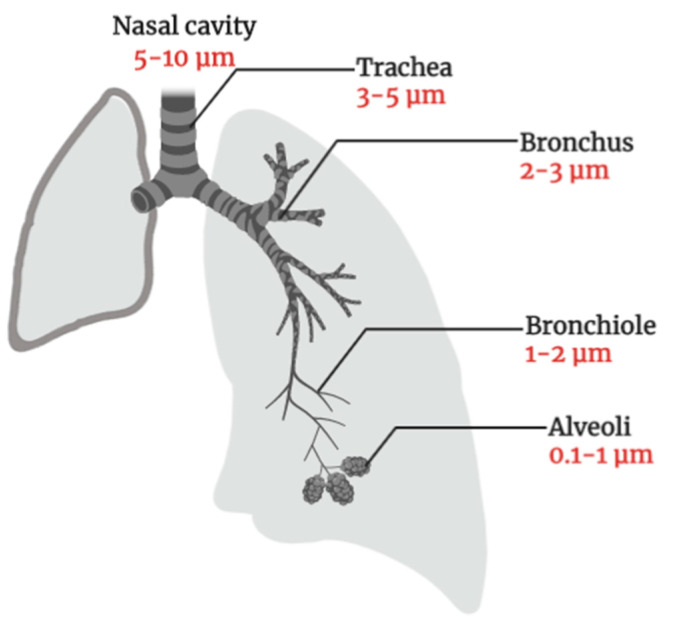
Pulmonary deposition of inhaled particles in healthy lung-dependent on the particle size (Illustrated through Biorender.com).

The pulmonary route is an attractive route of administration for lung cancer therapy as the lung has a thin absorption membrane (0.1–0.2 μm) which provides a large surface area for absorption (~100 m^2^), and a higher blood flow (5 L/min) that will distribute the drug molecules to the whole body instantaneously. To ensure the efficacy of pulmonary delivery, DCX must be able to reach to the targeted site. Considering the anatomy of the lung (Figure 3), a drug will be deposited at different regions based on its particle size [42]. Particles with a size larger than 10 μm will be retained at the oropharyngeal region and the larynx by inertial impaction. The particle with a size between 2 to 5 μm will be deposited in the tracheobronchial region. Meanwhile, the small size particles (0.5–2 μm) will be deposited in the respiratory zone (i.e., bronchioles, alveolar ducts and alveolar sacs) via gravitational sedimentation and particles smaller than 0.5 µm will be exhaled out [43]. 

In recent years, there have been many interests in developing DCX nanoparticles for IV and pulmonary delivery. In general, there are many potential advantages of nanoparticle drug delivery, which include improved drug serum solubility, prolonged systemic circulation, controlled release as well as targeted delivery [44]. However, the utilization of nanoparticles for pulmonary delivery is not straightforward. Since nanoparticles are in the nano range, the developed formulation is expected to be exhaled out. To overcome this limitation, the formulation can either be nebulized into colloidal suspension or mixed with micro-sized inert carriers (carbohydrates, amino acid or phospholipid) or embedded in microparticles. The formulation will also depend on the type of aerosol device intended to deliver the drug to the lung [45].

### 4.2. DCX Formulations 

#### 4.2.1. Taxotere^®^ Formulations

To overcome the solubility problem of DCX as mentioned earlier, the pioneer commercialized DCX formulation, Taxotere^®^, is formulated to contain 40 mg/mL of DCX with polysorbate 80 and ethanol for intravenous administration. Since its introduction in the market in 1996, Taxotere^®^ has been packaged in a set of two vials, where the first vial contains the concentrated formulation of DCX in polysorbate 80 and the second vial contains ethanol (95% *v/v*) as diluent. In 2010, Taxotere^®^ was marketed as a single vial that maintained the same docetaxel to polysorbate 80 ratios as that of the two vials [46]. Since the patent of Taxotere^®^ expired, several generic DCX formulations approved by the FDA such as Docefrez^®^ (SunPharma) and Docetaxel Accord (Accord Healthcare) as well as many other generic DCX formulations also include polysorbate 80 in the formulations [47].

Taxotere^®^ is associated with a variety of acute and long-term side effects including hypersensitivity, febrile neutropenia, fatigue, fluid retention, and peripheral neuropathy [48]. The occurrence of hypersensitivity reactions and fluid retention have been partly attributed to polysorbate 80 [49]. Polysorbate 80 can also inhibit the binding of taxanes to albumin [50], thus affecting the albumin-based drug transport [47]. The formulation also causes the decreased uptake by tumour tissue, and elevated exposure of other body compartments to the drug [51,52,53]. To overcome these issues, an alternative drug delivery system (DDS) without polysorbate 80 have been considered. Among the most popular alternatives is a nanoparticle-based drug delivery system.

#### 4.2.2. DCX-Loaded Nanoparticles (NPs)

The recent literature shows an upward trend in the utilization of NPs, a branch of nanotechnology, for the delivery of DCX. This is mainly due to the fact that NPs are easily manipulated to increase the efficiency of drug delivery as well as the bioavailability of DCX [54]. NPs are particles with a diameter size of 1–1000 nm and the term is used in general for many different shapes and sizes of nanovector structures. Owing to their high surface area to volume ratio, NPs can alter the basic properties and bioactivity of drugs. NPs can also facilitate the intracellular uptake of a drug due to their nanoscopic size. The ability of NPs to encapsulate a drug can improve the pharmacokinetic properties and biodistribution, decrease the toxicity, and increase the solubility and stability of a drug. It can also deliver an anticancer drug to a specific site while controlling its release [54,55]. There are few features of NPs that can be easily adjusted in designing the drug delivery system: the composition, size, shape and surface properties. This review will discuss the currently available DCX-loaded NPs for treatment of NSCLC.

#### 4.2.3. Solid Lipid Nanoparticles (SLNs)

**Figure 4:**
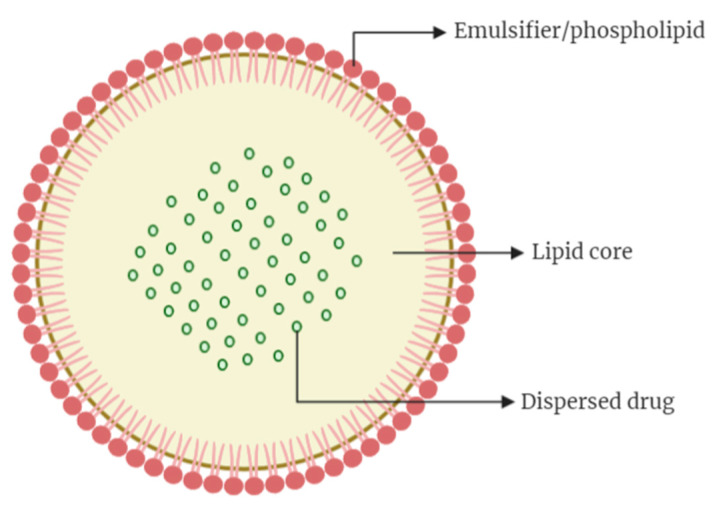
Structure of solid lipid nanoparticles (Illustrated through Biorender.com).

SLNs are colloidal drug carriers in the size range of 50–1000 nm and are prepared through dispersing melted solid lipid in water, in the presence of emulsifier(s). This type of NPs, which was first introduced in 1991, represents a more advanced vehicle for drug delivery in comparison to the colloidal vehicles such as emulsions, polymeric NPs, and liposomes [56]. SLNs usually consist of spherical solid lipid particles in water or aqueous emulsifier solution. The structures (Figure 4) are generally made up of solid hydrophobic core with monolayer or multilayer of phospholipid coating (emulsifier) and the core will contain the dissolved or dispersed drug. The hydrophobic chains of phospholipids are embedded in the fat matrix with the potential to carry a lipophilic drug, whilst the hydrophilic segment of the phospholipid stay outward to ensure ease of solubilization in aqueous vehicles. The encapsulation of hydrophilic drugs in a conventional SLN is a challenge due to incompatibility between hydrophilic molecules with the lipids and high leakage of the loaded drug into the surrounding aqueous environment. Attempts were made to prepare SLNs with the ability to load hydrophilic drugs by methods such as double emulsion technique and incorporation of different type of lipids, with different rates of success, as reported in the literature [57,58].

**Figure 5:**
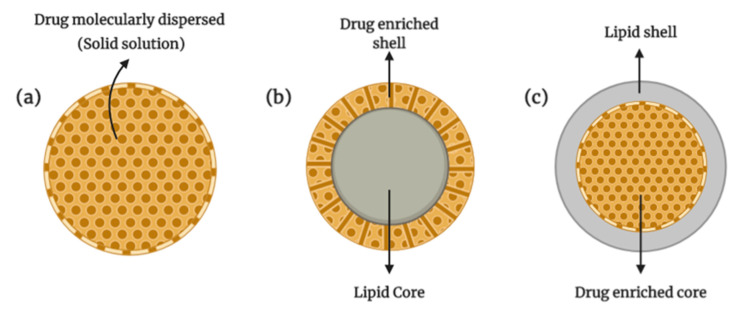
Structure of various models of incorporation of active compounds into SLNs: (**a**) solid solution (homogenous matrix) model, (**b**) drug-enriched shell model, (**c**) drug-enriched core model (Illustrated through Biorender.com).

In preparing SLNs, an emulsifier is used to stabilize the dispersion along with a wide range of lipids: lipid acids, mono-, di-, or triglycerides, and glyceride mixture or waxes. The lipids that made up the nanocarrier allowed SLNs to stay in solid form at room and body temperatures [59]. In order to stabilize the SLNs in dispersion, various surfactants are used to cover the surface of SLNs. The commonly used surfactants are non-ionic types, which includes Poloxamer 188, Poloxamer 407, Span and Tween. The common methods used to prepare SLNs are high-pressure homogenization and solvent emulsification, which provide highly lipophilic lipid matrix for drugs to be dispersed or dissolved into. The incorporation of a drug into SLNs can be done either by dispersing it homogenously in a lipid matrix, placing it into the shell surrounding the lipid core or incorporation into the core surrounded by the lipid shell (Figure 5). SLNs offer few advantages as DDS which include controlled drug delivery, good biocompatibility and biodegradability, improved bioavailability and higher stability. The lipids used in the production of SLNs are usually similar to physiological lipids, which gives their biocompatible characteristic. In addition, the production method that uses high-pressure homogenization is viable at the industrial scale, hence making SLNs a potentially useful and commercializable DDS [60,61].

SLNs have been widely used to deliver antitumor chemotherapeutic moieties as they can minimize the drawbacks of conventional chemotherapy. Due to their lipid core composed of biodegradable lipids, SLNs could reduce the risk of chronic and acute toxicity and at the same time enhance the therapeutic effectiveness of the encapsulated drugs. SLNs as a DDS are not without any limitations; one of them is the rapid elimination from the blood circulation by the reticular endothelial system (RES). This will limit the amount of drug delivered at the diseased cells. In addition, the system also suffers from a low drug loading efficiency due to the highly packed lipid crystal network. Under such conditions, the amount of drug molecules able to be incorporated will be reduced. In addition, the difficulty in solubilizing the drug molecules in the lipids used as the SLN materials will further complicate the drug loading issue [62]. This is indeed a significant problem and to overcome it, an improved system called ‘Nanostructured Lipid Carrier’ (NLC) is prepared, which incorporated liquid lipids into the solid lipids. This was found to produce a more flexible carrier, with more disrupted network within the particles, hence allowing the incorporation of more drug molecules [60].

To overcome the rapid RES clearing, it was proposed that the NPs can be coated with stable, biocompatible, and hydrophilic polymers such as polyethylene glycol (PEG), poloxamers, or poloxamines [63,64]. The most promising strategy in reducing RES uptake is to decrease the particles size and to sterically stabilize the NPs with a layer of amphiphilic polymer chains such as PEG. For example, Naguib et al. (2014) reported trimyristin-based PEGylated DCX-loaded SLN, which demonstrated higher cytotoxicity against various human and murine cancer cells in vitro compared to the DCX solubilized in Tween 80/ethanol solution. This formulation also showed a lower concentration of DCX in major organs such as liver, spleen, heart, lung, and kidney, indicating the ability of PEGylation of overcoming the RES clearance problems associated with SLNs [65]. 

The SLNs DDS designed for pulmonary delivery of DCX was reported by Li and colleagues. In their study, baicalein (BA) and DCX were incorporated in glyceryl monostearate (GMS) matrix with transferrin (Tf) and PEG-hydrazone [66]. The DCX-loaded SLN was prepared as a combination therapy as a strategy to overcome DCX resistance affiliated with drug efflux pump P-gp. BA possesses antioxidant and antitumor effects by prompting cell cycle arrest, controlling apoptosis and hindering the signal pathways. The synergistic activity of BA with cisplatin in inhibition of A549 lung cancer cells has previously been documented [67] using the Chou Talalay method. The combination index (CI) value was found to be less than 1 while the fraction of affected cells (Fa) value was between 0.2 and 0.8, indicating synergistic effect of both drugs in the SLNs formulation. In addition, the PEGylated SLNs showed a better release characteristic of the loaded DCX-BA compared to the non-PEGylated SLN, with a longer circulation time of the system in blood [66]. 

In addition to lung cancer, DCX-loaded SLNs for various cancer treatment have been reviewed by Sumera and co-workers [68]. In general, DCX-loaded SLNs can be used for its controlled and site-specific drug delivery and enhanced antitumor activity. As SLN comprises of lipids, the safety, the biocompatibility and biodegradability of the materials would be sufficiently accepted in vivo [69].

#### 4.2.4. Liposomes

Liposomes are another lipid nanocarriers that have been used in anticancer DDS for many years due to their advantages on enhanced drug delivery. Among the advantages are protection of the drug against environmental factors, improvement in the performance of the products, delayed degradation of the encapsulated drug, cost-effectiveness in the formulation of expensive drugs and their potential of reducing systemic toxicity [70,71]. Liposome was first discovered and described by Bangham et al. in 1960 [72] for application as a carrier in drug delivery and since then, there have been many studies to investigate this type of nanocarrier. 

Liposomes are spherical vesicles with aqueous core enclosed by lipid bilayers, where the polar head groups are orientated in the pathway of the internal and external of the aqueous phase. The particles usually have a size ranging from 30 nm to several micrometres. They can have a single bilayer, which makes them into small unilamellar vesicles (10–100 nm) or large unilamellar vesicles (>100 nm) depending on their size. For multilayer liposomes, or also known as multilamellar vesicles, the size of the particles can range from 0.5 nm to 10 µm. 

The bilayer membrane assembly of liposomes can be made of synthetic or natural lipids and this is the reason for the biodegradability and biocompatibility of liposomes. The design of liposome can vary according to their composition, size, surface charge and method of preparation [73]. Thin-film hydration is the most commonly used technique, as evidenced in the multitude of publications available in the literature. The preparation involves solubilization of the lipid components in an organic solvent such as ethanol, evaporation of the solvent by rotary evaporation and rehydration of the lipid film in water. Other preparation techniques such as the reverse-phase evaporation, freeze-drying, and ethanol injection have also been reported [74].

Since the first liposome-based formulation that contains doxorubicin—Doxil^®^ was released into the market in 1995 for the treatment of ovarian cancer and AIDS-related Kaposi’s sarcoma [75], there have been significant developments in liposome technology for DDS. The therapeutic effect of the encapsulated drugs can be enhanced by altering their pharmacokinetics and pharmacodynamics. Drugs with a different lipid solubility profile can be encapsulated in the liposomes such that the strongly lipophilic drugs can be entrapped proximately in the lipid bilayer, whilst the hydrophilic drugs can be encapsulated in the aqueous core. The drugs with a moderate partition coefficient (logP) will be partitioned between the lipid and aqueous phase, both in the bilayer and in the aqueous core. 

**Figure 6:**
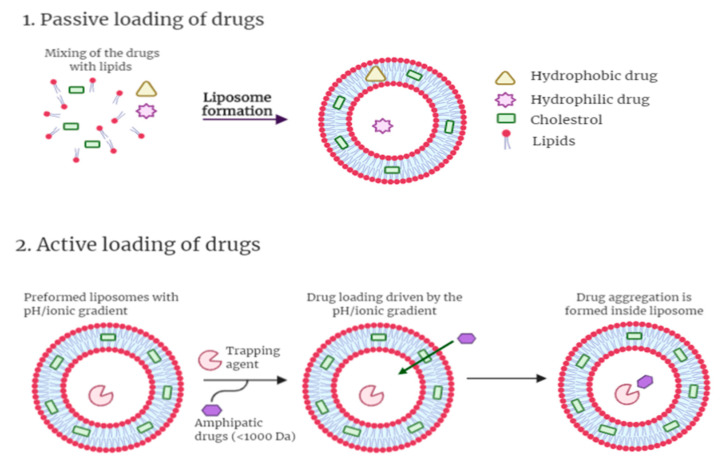
Illustration of liposome and its different drug-loading. The drug can be loaded in the liposomes by: (**1**) passive loading. The lipophilic drug is entrapped in the bilayers, and the hydrophilic drug is entrapped in the aqueous core. (**2**) Active loading where pH gradient method is applied (Illustrated through Biorender.com).

The mechanism of drug loading into liposome can be divided into passive and active loading methods. In the passive loading method, hydrophilic drugs will be distributed in the aqueous phase of the liposome and hydrophobic drug will be incorporated in the lipid bilayer simultaneously with the formation of the liposome. For hydrophobic drugs, an organic solvent is used to dissolve the drugs and lipids followed by solvent removal by evaporation to form a drug within a lipid thin film. The drug-containing thin film will then be hydrated with an aqueous phase to form liposome. For hydrophilic drugs, the lipid film will be dispersed in an aqueous phase containing the drug. In active loading method, pH gradient is used by preparing liposome with a low internal pH followed by the addition of base solution to the extra-liposomal medium. The amphipathic drug internalization into the liposome is driven by the transmembrane pH gradient. Once the drug permeates across the phospholipid bilayer(s), it will interact with the trapping agent such as ammonium sulfate, which produced a charged environment in the liposome. The drug would then diffuse into the liposome, interact with the sulfate ion, precipitate and lock inside the liposome with the trapping agent [76]. Both modes of drug loading are illustrated in Figure 6 below. 

Liposomes usually reach their action site by extravasation into the interstitial space from the bloodstream and it will stay in the tumour tissues due to its inefficient lymphatic system. The liposomes surface can be modified to improve its targeting ability by adding ligands on the outer surface of the lipid bilayer to actively target the tumour tissues. As an example, antibody-based approach by using immunoliposomes (ILP) can improve the specificity of liposomes to cancer cells or to the endothelial cells of tumour vasculature. Thermosensitive or pH-sensitive liposomes is another useful approach to ensure the specific release of the encapsulated drug at the targeted tumour cells [77,78].

As with other NPs, RES clearance is another important aspect to be looked into in the application of liposomes as DDS. There are several studies showing the importance of vesicle size, lipid composition, surface coating, surface charges, and liposomes–plasma protein interaction on the clearance of liposomes by the RES [79,80,81,82]. Hence, selecting suitable lipids, increasing the vesicle stability by adding cholesterol and coating the liposome with polymers (which will produce stealth liposomes) are the approaches that may improve the circulation time of liposomes in blood [83].

Pareira et al. (2016) formulated DCX-loaded liposomes (DCX-LP) in an effort to overcome DCX solubility and toxicity issues [84]. The effect of various compositions of liposome and the DCX:lipid ratio on the drug loading and steric stabilisation have been investigated. The lipids investigated include cholesterol (Chol), 1,2-distearoyl-sn-glycero-3-phosphoethanolamine-N-[amino(polyethylene glycol)-2000] (DSPE-PEG2000), and phospholipids (1,2-dioleoyl-sn-glycero-3-phosphocholine (DOPC), 1,2-dipalmitoyl-sn-glycero-3-phosphocholine (DPPC), 1,2-distearoyl-sn-glycero-3-phosphocholine (DSPC)). The unsaturated DOPC liposomes have the highest drug loading compared to the other rigid phospholipids (DPPC and DSPC). The steric stabilization showed minimal effect on DCX encapsulation into liposome. By decreasing the lipid to drug molar ratio (40:1 to 5:1), they found that the loading capacities of DOPC liposomes were enhanced, while for the DPPC and DSPC liposomes showed the contrary. The in vitro study conducted on PC3 cells also showed the ability of the DCX-LP to improve the poor tissue penetration of DCX, which implied improved therapeutic efficacy. This study shows that choosing the right composition of lipids is very important to ensure sufficient drug encapsulation, stability and therapeutic effect. The addition of cholesterol helps to stabilize the bilayer structure and improve the stability of the liposome. PEGylation, on the other hand, helped to stabilize the liposomes sterically and prolong their blood circulation.

In a recent formulation of DCX-LP by Arthur and co-workers, the authors reported the benefit of pre-treatment with telmisartan (TEL) to increase the uptake of the DCX-LP by the in vitro cell model and xenograft tumor models [85]. TEL is an anti-fibrotic agent that can inhibit cancer proliferation by reducing the transforming growth factor-β (TGF-β) signalling, as the TGF-β induced the epithelial-mesenchymal-transition (EMT) in cancer cells [86]. EMT induced by TGF-β in cancer cells (including NSCLC) was related to the resistance to apoptosis, stem cells traits acquisition, and chemoresistance [87]. EMT aided lung tumor in invading immune system by expressing Programmed Cell Death receptor ligand (PDL1) on the surface of the tumor, which later will interact with the Programmed Cell Death receptor protein (PD1). With the interaction of PDL1 and PD1, the activation of the T-cytotoxic cells against the solid tumor is hindered [88]. The efficacy of combination therapy of DCX-LP and TEL was investigated on mycoplasma free NSCLC cell lines (H460). The H460-WT (wild-type) cells studied through 3D analysis showed prominent improvement in the IC50 values of the tested DCX-LP in combination with TEL, as compared to the untreated liposome. Apart from that, in xenograft mice models, the anticancer activities by TEL and DCX-LP were shown by tumor volume reduction, apoptosis increment and cancer stem cells markers downregulation. This could be a future approach to improve the efficacy of liposomal-based formulation in cancer treatment. 

In another model of active targeting for DCX liposomes in lung cancer, a CD133 antigen with aptamer was conjugated with DCX-LP (CD133/DCX-LP) [89]. CD133 is a transmembrane glycoprotein and it is one of the commonly used cell surface antigens in the detection and isolation of cancer stem cells (CSCs) from various solid tumors, including the lung tumour [90]. In this study, CD133 antigen and A15 aptamer was used to target CD133-positive CSCs as these cells have a stronger ability in self-renewal, proliferation and differentiation as compared to CD133-negative CSCs [91]. DCX-LP formulation showed a sustained release profile owing to the presence of CD133 aptamer on the surface of liposome and higher internalization in A549 cell lines compared to free drug and non-conjugated DCX-LP. The antitumor efficacy of CD133/DCX-LP was shown by the anti-proliferative effect on A549 cell lines as well as higher tumor growth inhibition in tumor-bearing mice when compared with free drug and DCX-LP. The systemic toxicity of CD133/DCX-LP was also found to be on the lower side as indicated by the in vivo study. 

Targeting of liposomes with phospholipid-anchored folate conjugates has been long considered as attractive means for the delivery of chemotherapeutic agents including DTX to the folate receptors-expressing tumors [92]. For a localized therapy of lung cancer, inhalable dry powder DCX-LPs was decorated with folic acid (FA) through conjugation on the DCX-LPs surfaces [93]. The presence of FA will help to actively target folate receptor α (FR-α) that is overexpressed on the membrane of the tumor cells including NSCLC [94]. The FA-DCX-LPs prepared by thin-film hydration and spray dried with mannitol and leucine exhibited higher cellular uptake by SPC-A1 cells (lung cancer cell line with high expression of FR-α) which leads to a higher cytotoxicity against cancer cells as compared to non-spray dried FA-DCX-LPs formulation and free DCX. The therapeutic effect of spray-dried FA-DCX-LPs was higher, as indicated by biodistribution study in rats where 12 h post intratracheal administration, the drug accumulation in the lung was 25 times higher compared to intravenous administration at the same dose. The superior accumulation of the drug in lungs indicates that the accumulation of drugs in any other major organs such as heart, spleen, kidney and liver was lower, thus systemic cytotoxicity may be reduced. The effective targeting to cancer cells was achieved by conjugating FA to DCX-LPs which contributed to the enhanced therapeutic effect of DCX. Another study on DCX-loaded liposomes worth mentioned here is that by Mehendale and Athwale (2020). The dry powder inhaler of DCX-loaded liposomes contained lipids which were hydrogenated soy phosphatidyl-glycerol, cholesterol and disteroyl phosphatidyl-glycerol (sodium salt). The liposomes produced were lyophilized with trehalose as the cryoprotectant. A preliminary study on the A549 lung cancer cells showed promising results with the IC50 of 188 µg/mL [95].

#### 4.2.5. Polymeric Nanoparticles (PNPs)

Since first described by Langer and Folkman in 1976, PNPs have gained significant attention as a nanocarrier for DDS [96]. PNPs are synthesized from polymer. They are solid, nanosized (10–1000 nm) colloidal particles and the polymers used typically are biodegradable [97,98]. Depending on the method of preparation, two types of PNPs can be prepared, which are the nanocapsules and nanospheres. Nanocapsules are prepared by dissolving the drug in the liquid core of oil or water and a solid polymeric membrane encapsulates this core. In contrast, in nanospheres, the drug is incorporated in the polymer matrix. 

The method of PNPs preparation varies on the types of drug to be incorporated and the preference of the formulator towards a particular administration route. Among the common methods to prepare PNPs are solvent evaporation, solvent diffusion, nanoprecipitation and salting out. Other than nanocapsules and nanospheres, polymeric micelles, polymeric dendrimer, and polyplexes are regarded as polymer-based NPs [99]. The polymers used in the formulations of PNPs can either be from a natural source of polymer or synthetic polymer [99]. Natural polymers include different classes of polysaccharides such as chitosan, dextran, alginate, gelatine and albumin, which have the advantages of being biocompatible and biodegradable. The development of PNPs from biodegradable synthetic polymers has also gained attention due to their flexibility in the design of the PNPs, in addition to their favourable physicochemical properties over natural polymer. The synthetic polymers usually used in PNPs are poly (lactic acid) (PLA), poly (Lactide-co-Glycolide) PLGA and polycaprolactone (PCL) [100]. These synthetic polymers have been recognized by the FDA as Generally Regarded as Safe (GRAS), which allows their potential application into human use [101]. PNPs offer some advantages over other NPs such as stability in storage [102], higher drug loading especially for drugs with low solubility, homogenous particle size distribution, and longer circulation time [103].

For a better targeting DDS, biodegradable polymers can be engineered and functionalized to reach the tumor site more selectively. The sensitivity of these PNPs toward a certain environmental factors such as pH, redox potential, temperature, enzyme, light, and magnetic field could help to ensure the release of encapsulated drugs at the target sites [104]. The ability of the NP in general to target the leaky environment of the cancerous cells through enhanced permeability and the retention (EPR) effect would be augmented by the stimuli-responsive drug release.

A simple PNPs system with hydrophobic L-phenylalanine-poly (ester amide) (Phe-PEA) has been developed to improve the antitumor efficacy of DCX to suppress NSCLC by Chen and co-workers [105]. The DCX-Phe-PEA PNPs were prepared via nanoprecipitation method with a various composition of diacid and diol segments with different alkyl chain. As the alkyl chain length increased, the hydrophobicity also increased and led to an increase in the loading of DCX into the PNPs. The average particle size of the PNPs was about 100 nm with a loading capacity of 20% (*w/w*) and it showed low burst effect and sustained drug release in vitro. The in vivo study using BALB/c mice bearing A549 adenocarcinoma cells showed a better therapeutic effect as compared to blank PNPs, phosphate saline buffer, and Taxotere^®^. The longer circulation time of DCX-Phe-PEA NPs also contributed to this, allowing ample time for the DDS to reach the tumor site, leading to the reduction of cell proliferation, prevention of the metastasis, elevation of apoptosis and inhibition of the overall tumor growth. 

In another study, the PNPs loaded with DCX was developed for the treatment of lung cancer by inhalation [106]. The PNPs were composed of cholesterol-PEG co-modified poly(n-butyl) cyanoacrylate NPs (CLS-PEG NPs) loaded with DCX for sustained pulmonary delivery in cancer metastasis. The CLS-PEG NPs prepared through the emulsion polymerization method and spray-dried into a powder were then evaluated for the in vitro aerodynamic assessment. In addition, the pharmacokinetics analysis, tissue distribution analysis, and in vivo antitumor efficacy were also determined by using an orthotopic mouse model. The study showed that the DCX-CLS-PEG NPs had a high encapsulation efficiency of 96% and the drying method did not affect the encapsulation efficiency as well as a drug loading percentage. The encapsulated drug was released in a sustained manner whereby it achieved about 80% of DCX release after 24 h. 

In their pharmacokinetics study, they proved that the inhalation route is better than intravenous administration as the inhalation formulation showed the longer plasma concentration of DCX in rats’ lungs after intratracheal instillation. The inhalable form of the NPs improved the lung retention of the drug by about 4-fold compared to the free drugs. Other than sustained released and prolonged pulmonary absorption time, the inhalation formulation efficiency contributed by the fact that it can pass through the air-blood barrier in the lung, showing that the administration was non-invasive. Hence, the inhalable DCX PNPs have a high potential as useful DDS to treat lung cancer [106]. 

To achieve active targeting of a PNPs, Patel and co-workers (2018) conjugated a monoclonal antibody (cetuximab) on the surface of DCX-loaded PLGA NPs to target the NSCLC with overexpressed epidermal growth factor receptor (EGFR) [107]. Cetuximab (CET) will act as a tyrosine kinase inhibitor and bind to EGFR to inhibit the growth of the tumor cells and the division of the cancerous cells ([108]. The formulation of CET-DCX-PLGA NPs showed a more efficient antitumor effect as compared to free DCX and DCX-PLGA NPs as investigated in vitro and in vivo. The in vitro study on the A549 cells line showed that CET aided the DCX-PLGA NPs in cell internalization to tumor cells, sustained drug released, higher cellular uptake by the A549 cells, higher apoptosis rate of the A549 cells and these led to higher antiproliferative activity of CET-DCX-PLGA NPs. These characteristics also contributed to a high tumor inhibition growth in tumor-bearing mice and the weight loss of the mice was mitigated by CET-DCX-PLGA NPs. Based on these results, CET-DCX-PLGA showed that active targeting of PNPs could enhance the antitumor activity of the drug.

Another effective way to actively target lung cancer cells was shown by Chi et al., in which they developed a DCX-loaded PLGA NP conjugated with platelet membrane (PM) [109]. PM was chosen as targeting agent as it can prolong the circulation time of the carrier, it also possesses multiple adhesions molecules (i.e., glycoprotein Ib-IX-V, glycoprotein VI, C-type lectin-like-2-receptor, P-selectin and six different integrins) to selectively bind to tumor cells [110], and immune escape capabilities by decreasing the RES clearance [111]. The in vitro release study showed that PM-DCX-PNPs has the slowest release in A549 cell lines compared to free DCX and DCX-PNPs and the cytotoxicity study on A549 cells line also showed that PM-DCX-PNPs significantly inhibited the cell growth. Thus, this supports the hypothesis that PM can prolong the circulation time as DCX was released the slowest and the tumor-targeting efficacy was better due to the presence of PM, as indicated in the cytotoxicity study. Furthermore, the in vivo study also concur with the finding of the in vitro study. The in vivo study showed that DCX was released the slowest even after 24 h after administration of PM-DCX-PNPs as compared to DCX-PNPs and free drug. Moreover, PM-DCX-PNPs have the highest rate of tumor growth inhibition and the mice did not suffer from any significant weight loss. In this study, conjugating PM on the surface of the DCX-NPs not only improves its targeting ability to the tumor cell, but also overcome the low solubility and toxicity of DCX. 

A study by Wang et al. (2019) exploited the benefits of mesenchymal stem cells (MSC) such as its known anti-tumorigenic effects [112] and low immunogenicity [113] to be used in DCX loaded PLGA-*b*-PEG copolymer NPs for lung cancer targeting [114]. Another reason for utilizing MSC is because it was mostly trapped in the lung after intravenous administration in cell therapy [115]. In the in vitro study on A549 cell lines and in vivo study showed that MSC aided the NPs to target the lung cancer cells as confirmed by imaging. The in vivo study where DCX was loaded into MSC-PLGA-*b*-PEG NPs showed that the MSC could improve the antitumor activity as it inhibits tumor growth more efficiently than DTX-PNPs at a lower dose of DCX. 

Other than synthetic polymer, naturally occurring polymer has also been used to produce PNPs for drug delivery to lung cancer. Human serum albumin (HAS) was used by Qu et al. to prepare PNPs loaded with DCX [116]. The in vitro study showed that DCX-PNPs exhibited sustained release of DCX, better anti-proliferative activity against A549 cell lines and high cellular uptake. Together with the in vivo study, they confirmed that the prepared DCX-NPs had a low systemic toxicity towards mice as the maximum tolerated dose was higher than the free DCX. In another study, DCX-loaded chitosan NPs was developed for the treatment of various types of cancer [117]. The characterizations, in vitro and in vivo studies showed that the chitosan NPs had desirable results for drug delivery purpose. The in vitro study showed that DCX was released for over 24 h in a sustained manner and the in vivo showed that the NPs were biocompatible with low toxicity. These studies showed that natural polymers could also be a promising choice of material to develop nanoparticulate DDS for the treatment of lung cancer. 

#### 4.2.6. Polymeric Micelles (PMs)

Polymeric micelles (PMs) are made up of amphiphilic molecules (surfactants or block copolymers) that self-assemble in water above their critical micellar concentration (CMC). PMs have hydrophobic core and hydrophilic shell which make up the core/shell structure in the size of 10–100 nm [118]. As PMs is formed thermodynamically, once the concentration of amphiphilic molecules reaches below the CMC, the structure will be disrupted and the molecules will be dissolved in water [119]. The structure of PMs is suitable as a carrier for anticancer drug delivery. The core of PMs can entrap hydrophobic drugs, allowing a controlled drug release, while the hydrophilic shell can prevent the uptake of PMs by the RES, allowing a longer circulation time in blood. A longer circulation time will increase the likelihood of PMs accumulating in the tumor site by EPR effect, thus improving the bioavailability and therapeutic effectiveness of a drug. 

Although PMs, in general, possess the ability to accumulate at the tumor site, different production methods have been shown to produce PMs with different accumulation behaviours. In one study, DCX-loaded PLGA-PEG-maleimide (Mal)-based micelle produced using the microfluidic method showed a greater antitumor efficacy and higher accumulation rate at the tumor site, compared to the same micelles produced using the dialysis method [120]. The improvement was attributed to a smaller particle size and narrower size distribution of micelles produced using the microfluidic method which prompted a better uptake in the A549 cells. This study highlighted the advantage of using a microfluidic approach compared to the bulk approach (i.e., dialysis method).

Apart from the ability of micelles to do passive targeting (i.e., EPR effect), an active targeting was also explored in a recent study [121]. The study used octreotide (OCT) to modify the surface of curcumin (CUR)/DCX-loaded micelles to prevent tumor metastasis in NSCLC. OCT acted as a targeting ligand to somatostatin receptor that is overexpressed on tumor cell membranes. The in vitro study showed that OCT enhanced the cytotoxicity and cellular uptake of the micelles via receptor mediated endocytosis. The in vivo study on A549 tumour-bearing BALB/c mice supported the in vitro results as the micelles showed better distribution in the tumor site due to both active and passive targeting. The micelles also exhibited the strongest inhibitory effect on tumor volume and weight of the treated animals. 

In another study by Gong et al. (2020), DCX was loaded into *N*-(tert-butoxycarbonyl)-*L*-phenylalanine end-capped methoxy-poly (ethylene glycol)-block-poly (*D, L*-lactide) (mPEG-b-PLA-Phe(Boc)) micelles (DCX-PMs) [122]. The formulation showed an improvement in the aqueous solubility of DCX, from 0.006 to more than 10 mg/mL, a ~1600-time increase. Moreover, it also showed a better stability due to the strong interaction between Boc-*L*-Phe and DCX, giving an advantage of logistics and clinical usage. A less toxic and efficient formulation of DCX was possible as the in vivo study of DCX-PMs on A549 tumor-bearing xenograft model showed a better inhibition in tumor growth and better biocompatibility in the mice compared to Taxotere^®^. The in vivo pharmacokinetic distribution study also demonstrated sustained release of DCX-PMs at the tumor site, thus making PMs another efficient nanocarrier to deliver DCX in lung cancer therapy. 

#### 4.2.7. Lipid-Polymer Hybrid Nanoparticles (LPHNPs)

Lipid-polymer hybrid nanoparticles (LPHNPs) is another versatile DDS as it possesses the advantages of both liposomes and polymeric NPs. This DDS has successfully overcome limitations such as NPs structural disintegration, limited circulation time, and pre-mature content release [123]. This system can deliver drug either through active targeting or passive targeting.

**Figure 7:**
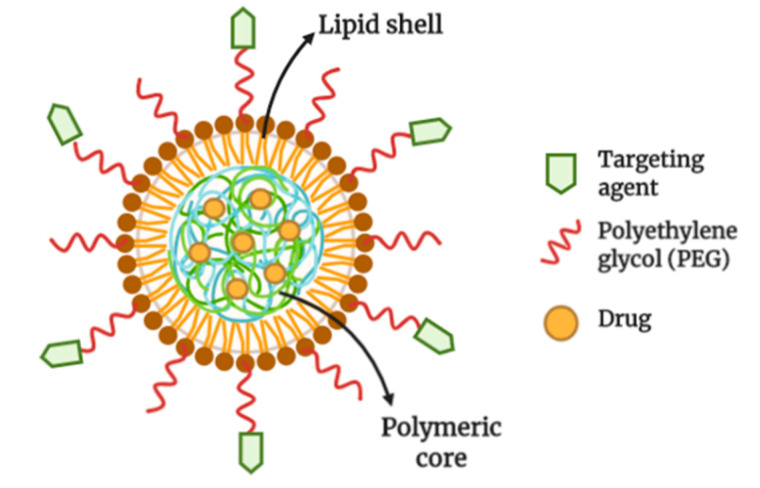
Structure of lipid-polymer hybrid nanoparticles (LPHNPs). LPHNPs is made up of polymeric core loaded with a drug(s). The polymeric core is surrounded by a lipid/lipid-polyethylene(glycol) (lipid-PEG) monolayer. The nanoparticle is functionalized by conjugating ligands onto the PEG (illustrated through Biorender.com).

In general, the nanocarrier system composed of a central core made up of polymer and drug or the drug alone, a middle layer made up of lipid to protect the polymer and an outer layer made up of lipid-coated with PEG for steric stabilization (Figure 7). The system can be formulated using a one-step method or two-step method. In the latter, the PNPs are first prepared using either nanoprecipitation, emulsion-solvent evaporation, or high-pressure homogenization methods. The lipid is then integrated within the PNPs by directly adding the PNPs to a dried lipid film or by adding the PNPs into a lipid vesicles that are prepared by thin-film hydration method [124]. On the other hand, the one-step method only requires a mixing of lipid and polymer, which lead both materials to self-assemble. 

In a recent study, a selective targeting of LPHNPs was explored by conjugating the carrier with aptamer (APT) to deliver cisplatin (CDDP) and DCX for combination therapy of NSCLC [125]. Prior to drug loading into the NPs, DCX is conjugated with glyceryl monostearate (GM) to produce a redox-sensitive DCX prodrug (DCXp). In the study, DCX was released faster in hypoxic condition owing to the redox-responsive DCXp. The uptake of the APT-DTXp/CDDP-LPHNPs was higher than NPs without APT, as APT can selectively bind and internalized by the A549 cells. In addition to the selective targeting, synergistic combination of CDDP and DCX showed a better tumor inhibition ability in lung cancer xenograft mice, when compared to PAT-free LPHNPs and single drug-loaded LPHNPs.

In addition to APT, conjugation of LPHNPs with epidermal growth factor (EGF) was also studied to target the endothelial growth factor receptor (EGFR) that are overexpressed on NSCLC cells [126]. Targeting a particular receptor is important as about 10%–15% NSCLC patients in America and Europe and 50% of NSCLC patients in China have EGFR mutation [127]. In the study, the EGF LPHNPs that were loaded with DTX and resveratrol (RSV) showed a high encapsulation efficiency and sustained release of both drugs. The presence of EGF caused an increased uptake of the EGF LPHNPs in HCC827 cells that overexpresses EGFR as compared to HUVEC cells that has no expression of EGFR. The EGF DCX/RSV LPHNPs showed a greater inhibition in HCC827 and NCI-H2135 NSCLC cells compared to the DCX/RSV LPHNPs and free drugs. The EGF DCX/RSV LPHNPs also showed a better antitumor activity compared to free drugs and DCX/RSV LPHNPs using a lung cancer-bearing mice model, as indicated by the lower tumor volume, higher tumor growth inhibition ratios and absence of weight loss in mice. Thus, the formulation showed its potential to be used in the treatment of NSCLC due to its greater antitumor efficacy, synergistic activity and low systemic toxicity as it was conjugated with EGF to achieve its active targeting.

### 4.3. Inorganic Nanoparticles

#### 4.3.1. Carbon Nanotubes (CNTs)

**Figure 8:**
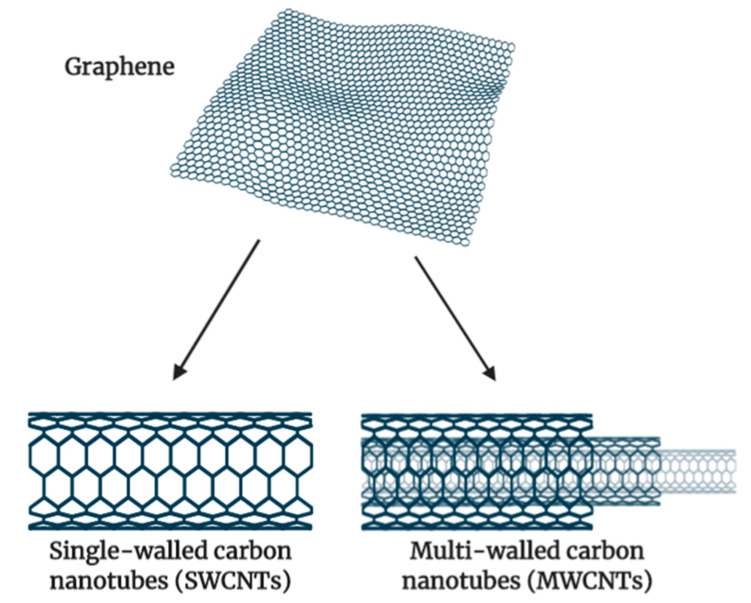
Structure of carbon nanotubes as originated from a graphene sheet. A graphene sheet is rolled to form SWCNT, and multiple sheets of graphene with different sizes are needed to form MWCNT (Illustrated through Biorender.com).

CNTs belong to the family of fullerenes and consist of a layer of graphite rolled up into a cylinder. CNTs are allotropes of carbon with a nanostructure that can be measured to have a length-to-diameter ratio greater than 1 million [128]. CNTs can be divided into two types: single-walled carbon nanotube (SWCNT) and multi-walled carbon nanotube (MWCNT). The former consist of one sheet of graphene rolled up to form a tube while the latter comprised of several concentric graphene sheets rolled into a tube [129]. The structure of both SWCNT and MWCNT are illustrated in Figure 8 below. 

CNTs exhibit some unique physicochemical and biological properties that make them a promising carrier in drug delivery for cancer therapy. Their tumor-accumulating properties and ability to cross the cell membrane barrier lead to an improvement in the delivery of therapeutically active ingredients [130]. CNTs has gained interest among researchers due to their nano-needle shape, hollow monolithic structure, high surface area, ultralight weight and their availability for surface modification [131,132]. Due to their high surface area, CNTs are capable of adsorbing and conjugating with therapeutic molecules. The surface modification or functionalization can enhance CNTs’ dispersibility in the aqueous phase as well as providing functional groups that will bind to desired therapeutic materials. 

Few studies have explored conjugation of MWCNT with several bioactive molecules (i.e., drugs, surfactant, diagnostic agents, antibody, targeting agents) that can target overexpressed receptors on the cancer cells [133,134,135,136,137]. In one study, conjugation of MWCNT with transferrin showed a better targeting of DCX against the A549 cell line [138]. The formulation showed an active targeting towards transferrin receptor that is overexpressed on cancer cells, resulting in a better in vitro efficiency and in vivo safety compared to commercialized DCX injection (Docel^TM^). Combination of a few strategies is also possible as shown in a study by Li and co-workers [139]. The study reported a formulation of SWCNT that was pH-responsive, conjugated with arginylglycylaspartic acid (RGD). The RGD peptide was shown to selectively bind to the surface receptor of the A549 cells, promoting the transport of DCX-loaded SWCNT. Additionally, the system also showed a higher release of DCX in lower pH which corresponded with microenvironment pH of tumor tissue and good safety on an animal model.

#### 4.3.2. Mesoporous Silica Nanoparticles (MSNs)

MSNs falls under ceramic NPs which have gained attention in research due to their biocompatibility, ease of synthesis and surface modification. Furthermore, MSNs also have other unique properties, such as their tuneable size and morphology, tailored mesoporous structure, uniform tuneable pore size, high chemical and mechanical stability, high surface area and pore volume as well as high drug loading [40,140,141]. With the ease of surface functionalisation, enhancement of therapeutic efficacy and toxicity reduction of a drug are achievable using MSNs [142]. MSNs typically have diameter in a range of 50–200 nm with a narrow particle size distribution and pore dimension of 3–4 nm [143]. MSNs can load a large amount of drug and their nanosized properties can assist in their accumulation on tumorous cell. 

In a study by Dilnawaz and Sahoo (2018), co-delivery of carfilzomib with anticancer drugs (i.e., etoposide or DCX) was attempted using MSNs along with the delivery of surviving siRNA [144]. The study showed a higher gene silencing efficiency of the system in A549 cells as compared to combined drug-loaded MSNs and combined native drugs. Furthermore, surface functionalization of MSNs was also possible using linkers that are specifically cleaved by matrix metalloproteinase (MMP9), to selectively target high-expressing MMP9 and tumor areas [145]. In the study, the cisplatin-loaded system demonstrated a selective targeting and caused significant apoptosis in human lung tumor ex vivo tissue culture application (3D-LTC), but not in healthy human lung tissue 3D-LTC. Although the study was not done on the delivery of DCX, the application of the study can be extended to improve the delivery and safety of DCX in the treatment of lung cancer.

#### 4.3.3. Gold Nanoparticles (AuNPs)

In the detection and direct cancer therapy, either with or without drug loaded into the AuNPs, gold has been one of the most popular choices [146]. AuNPs are stable colloid solutions of Au atom clusters with particle dimension range of 1 nm to 100 nm. AuNPs can also be synthesized into various shapes, such as spheres, rods, quantum dots, multipods, cubes, nanoclusters, nanofibers, stars, or hollow structures (i.e., shells, tubes, cages, or boxes), depending on the application of the AuNPs [147]. AuNPs offer advantages such as a strong optical absorbance that is optimal for detection and photothermal properties, making it a suitable option for anticancer therapy [148]. The DDS also possess outstanding physical and chemical properties, such that they are safe, stable and easy to prepare, have a small size, high surface area, quantum size effects and electrical effects [149,150]. The surface of the AuNPs can be easily modified by amine and thiol groups for tumour specific targeting [151]. Th encapsulation efficiency of AuNPs can be enhanced by conjugating the drug molecules to the surface or the structures of AuNPs with hollow interiors. The system can also be tailored for controlled release by adding a layer of thermo-responsive polymers on the surface AuNPs.

In a study by Thambiraj et al. (2019), DCX and folic acid (FA) were conjugated to preformed AuNPs for lung cancer delivery [152]. The DCX-loaded AuNPs/FA demonstrated specificity towards a lung cancer cell line (i.e., H520), shown by a 50% decrease in cell survival when compared with DCX alone. The specific targeting may be contributed by the FA whose receptors are overexpressed on the lung cancer cells.

## 5. Perspective

With advances in nanotechnology, various research studies are ongoing to find arsenal treatments for cancer whilst making it more convenient for the patients. Treatment of lung tumors such as NSCLC remains a significant clinical challenge in which the current standard treatment with chemotherapy and surgery are relatively challenging. Although newer drugs that target different histological subtypes and driver mutations were introduced (e.g., tyrosine kinase inhibitors), DCX remains the most potent drug in the treatment of lung cancer.

Various nanoparticulate formulations were explored in the quest for reducing DCX-related toxicity and manufacture a slow-release system. Since nanoparticle formulations generally have their advantages of passive targeting (i.e., through EPR effect), especially to the tumor site, recent approaches heavily focused on enhancing the delivery of DCX through active targeting. Active targeting was achieved by surface modification or functionalization of the nanoparticle using a substrate of receptors that are overexpressed in lung cancer cells (e.g., folic acid, somatostatin). Other strategies of using redox-sensitive DCX prodrug and pH-responsive SWCNTs will ensure that DCX will be in its active form in conditions that suit the tumor microenvironment. These approaches may be able to reduce the drug uptake in normal cells, thus reducing toxicity associated with DCX (e.g., mouth sores, hair loss). Furthermore, NPs such as SLNs, PMs, and LPHNPs can offer the advantage of a hydrophilic surface which allow longer circulation time in blood, giving enough time for the active and passive targeting to take place. Although the active and passive targeting showed enhancement in the efficacy of DCX, it is limited to improving the delivery of the drug to the target site which resulted in an improved uptake into cancer cells. In an effort to further enhance the cytotoxicity of the drug towards the lung cancer cells, few researchers have attempted to combine DCX with other compounds (e.g., siRNA, polyphenol, flavonoid) for synergistic activity, as mentioned earlier in the article. Due to the different targeting in the cellular pathway, the combination may also be effective on DCX resistance cell lines. This strategy combined with active and passive targeting would create an ideal remedy for DCX delivery. There are also many studies on the production of inhalable NPs for the delivery of DCX. This means that inhalation will be a future avenue to improve the specificity of the delivery and to reduce the side effect of the drug. However, more evidence and detailed studies will be needed before this type of formulation can enter the clinics.

In the scope of DCX delivery for lung cancer therapy, some NPs have been widely explored while some (e.g., AuNPs) have not. To our knowledge, only one study has been carried out in exploring active targeting of AuNPs/FA to deliver DCX. The AuNPs may be an interesting carrier to be used for delivery of DCX, as there have been many studies reported on AuNPs’ potential in cancer treatment with other drugs [153,154]. AuNPs can be further developed for theranostics due to the high atomic number of Au, which provides large X-ray absorption cross-section and photothermal conversion ability. Furthermore, due to these unique properties, AuNPs has been widely used for radiotherapy, photothermal therapy and photodynamic therapy as compared to any other inorganic metal in cancer treatment. 

## 6. Conclusions

This review summarized the current nanotechnology approaches in drug delivery systems that were developed for the passive and active delivery of DCX with different routes of administration and types of nanocarriers for the treatment of lung cancer. We hope this will open a new window for research into the nanoparticulate system for the delivery of DCX. Though the nanoparticle formulation development and preclinical assessment are in the superior stage, clinical trials are significantly lagging. This could be due to the lack of acceptance by physicians, owing to safety concerns and practicality (in term of cost and logistics) of the medication for treating the cancer patients. Hence, if these hurdles are mitigated satisfactorily, the DCX-incorporated nanoparticulate system certainly has the potential for cancer treatment. Perhaps, a constructive collaboration between multinational pharmaceutical companies and global organizations could make the DCX nanoparticles dosage form a reality to combat cancer.
